# Advanced practice physiotherapy in patients with musculoskeletal disorders: a systematic review

**DOI:** 10.1186/1471-2474-13-107

**Published:** 2012-06-21

**Authors:** François Desmeules, Jean-Sébastien Roy, Joy C MacDermid, François Champagne, Odette Hinse, Linda June Woodhouse

**Affiliations:** 1 School of Rehabilitation, Faculty of Medicine, University of Montreal, Montreal, Quebec, Canada; 2 University of Montreal Public Health Research Institute, University of Montreal, Montreal, Quebec, Canada; 3 Maisonneuve-Rosemont Hospital Research Center, University of Montreal Affiliated Research Center, Montreal, Quebec, Canada; 4 Department of Physical Therapy, Faculty of Rehabilitation Medicine, University of Alberta, Edmonton, Canada; 5 Department of Rehabilitation, Faculty of Medicine, Laval University, Quebec City, Quebec, Canada; 6 Centre for Interdisciplinary Research in Rehabilitation and Social Integration, Quebec City, Canada; 7 School of Rehabilitation Science, McMaster University, Hamilton, Quebec, Canada; 8 Hand and Upper Limb Centre, London, Quebec, Canada; 9 McCaig Institute for Bone and Joint Health, Calgary, Alberta, Canada

## Abstract

**Background:**

The convergence of rising health care costs and physician shortages have made health care transformation a priority in many countries resulting in the emergence of new models of care that often involve the extension of the scope of practice for allied health professionals. Physiotherapists in advanced practice/extended scope roles have emerged as key providers in such new models, especially in settings providing services to patients with musculoskeletal disorders. However, evidence of the systematic evaluation of advance physiotherapy practice (APP) models of care is scarce. A systematic review was done to update the evaluation of physiotherapists in APP roles in the management of patients with musculoskeletal disorders.

**Methods:**

Structured literature search was conducted in 3 databases (Medline, Cinahl and Embase) for articles published between 1980 and 2011. Included studies needed to present original quantitative data that addressed the impact or the effect of APP care. A total of 16 studies met all inclusion criteria and were included. Pairs of raters used four structured quality appraisal methodological tools depending on design of studies to analyse included studies.

**Results:**

Included studies varied in designs and objectives and could be categorized in four areas: diagnostic agreement or accuracy compared to medical providers, treatment effectiveness, economic efficiency or patient satisfaction. There was a wide range in the quality of studies (from 25% to 93%), with only 43% of papers reaching or exceeding a score of 70% on the methodological quality rating scales. Their findings are however consistent and suggest that APP care may be as (or more) beneficial than usual care by physicians for patients with musculoskeletal disorders, in terms of diagnostic accuracy, treatment effectiveness, use of healthcare resources, economic costs and patient satisfaction.

**Conclusions:**

The emerging evidence suggests that physiotherapists in APP roles provide equal or better usual care in comparison to physicians in terms of diagnostic accuracy, treatment effectiveness, use of healthcare resources, economic costs and patient satisfaction. There is a need for more methodologically sound studies to evaluate the effectiveness APP care.

## Background

Over the past few decades the convergence of rising health care costs and physician shortages have made health care transformation a priority in many countries [1,2]. These health system transformations have resulted in the development of new models of care that have been the impetus for legislative changes to enable health care practitioners to practice to their maximum scope. Such initiatives have demanded innovation and close collaboration among health care providers and have resulted in the emergence of new interprofessional models of care that often involve the extension of the scope of practice for allied health professionals [3,4]. For professionals suchs as nurses, these models of advance practice have however existed since World War I and later evolved into what is known today as Nurse Practitioners/Advanced Practice Nursing roles or for physiotherapists, primary care roles also expanded in wartime with formalization of extended scope/advanced practice roles in the United States during the Vietnam War. But today, aging of the population and increased prevalence of chronic diseases is taxing health care systems thus demanding broader implementation and expansion of such models [5]. Formal evaluation of these new models is necessary to insure timely access to efficacious and effective health care services. Evaluations involve a variety of different research designs to assess structure, process and outcomes related to these new models of care. These include evaluation of the extended scope practitioner’s competencies (e.g. diagnostic accuracy) and satisfaction with their new roles, the patient’s satisfaction with the new service delivery models, cost of the new models of care and whether they have improved outcomes (such as reduced wait times, expedited recovery and improved function for the recipients of the care).

Physiotherapists have emerged as key providers in such new roles, especially in settings providing services to patients with musculoskeletal disorders [6]. Many countries now report implementation of physiotherapists in what are called “advanced practice” or “extended scope practice” roles [7-11]. The new roles include role enhancement and role substitution related to traditionally performed medical or controlled acts, such as: communicating a diagnosis, triaging potential surgical candidates, ordering diagnostic imaging or laboratory tests, and prescribing/injecting medication. The new models of care involving advanced physiotherapy practice (APP) are ultimately aimed at improving access to care, with equal or better effectiveness, while containing costs and retaining patient and other health care provider satisfaction [8,12,13]. The majority of these initiatives have been implemented in emergency departments and orthopaedic clinics for the treatment of patients with common musculoskeletal disorders. Evidence of the systematic evaluation of the APP models is scarce, with the majority of reports being solely descriptive in nature [8]. Systematic review and structured methodological quality appraisal of the quantitative studies is notably lacking. Two reviews of the role of advanced practice physiotherapists (APPs) in different settings, and with various clienteles, have been published previously [8,11]. The first review published in 2006 focused on advanced practice roles in 5 allied health professions, including physiotherapy. The authors noted that only 7 studies evaluating APP roles published before 2005 were found to be methodologically sound following assessment using a quality appraisal tool. However, 6 of the studies included in that review presented only descriptive quantitative data or used a qualitative design [11]. The remaining studies of 145 APPs that did not present any quantitative data, or were found to have methodological limitations, were reviewed in another study by the same authors [8]. The authors concluded that although the majority of studies had methodological limitations, the role of the APP was beneficial, particularly in terms of access to care and patient satisfaction. The vast majority of the included studies of APP roles were focused on the management of patients with musculoskeletal disorders. The authors highlighted insufficient data in the literature regarding the safety and efficacy associated with the new APP roles [8,11].

A more recent review of the role of APP services included eleven studies that examined the effectiveness of advanced practice/extended scope physiotherapists working within emergency departments in primary care roles. This review included studies up to March 2009, however only 7 of the 11 studies referred to an advanced practice role and 3 of these studies were only descriptive in nature. Since the publication of these reviews, new studies evaluating the roles of APPs have been published. The aim of the current systematic review was to update the evaluation of the expanding role of advanced practice/extended scope physiotherapists in the management of patients with musculoskeletal disorders.

## Methods

### Literature search and study identification

A search in three databases, Medline, CINAHL and Embase, was performed using a modified search strategy based on the keywords that Kersten et al. [8] used in their systematic review. Their original literature search involved a three-part search strategy framework that included: (a) professions (physiotherapy), (b) intervention (advanced practice) and (c) outcome (for patients, other health professionals working with APPs, and health services delivery). This comprehensive search strategy used a combination of MeSH terms (subject headings) and keywords for professions (physiotherapy) and interventions (APP). Kersten et al. have published elsewhere the full literature search strategy [8]. We also added three new components to the present search strategy and included: 1- keywords related to diagnostic ability and/or diagnostic agreement between APP and other health providers or diagnostic imaging; 2- keywords related to emergency medicine and 3- the search was limited to a population with musculoskeletal disorders (Appendix). Manual searches of previous published reviews and retrieved study reference lists were also conducted. The review included articles published between 1980 and November 2011.

### Data extraction and quality assessment

#### Study selection

Abstracts of each article were reviewed by two authors (FD and LJW) to determine eligibility. Pairs of raters (FD and LJW or FD and JSR) then independently reviewed each article to determine whether it met the following inclusion criteria: 1) related to physiotherapy and advanced practice defined as new roles for physiotherapist that include role enhancement or role substitution related to traditionally performed medical or controlled acts, 2) addressed the impact or the effect of advanced practice in its broadest sense including competency studies evaluating diagnostic accuracy or ability to correctly triage, 3) written in French or English, 4) included patients with musculoskeletal disorders, 5) the article presented quantitative original data where APP care is compared to usual care or APP diagnostic is compared to another reference standard (imaging modality or another practitioner diagnostic). Descriptive studies were not included but studies of any other designs were included regardless of the outcomes measures used.

#### Methodological quality appraisal tools

Analysis of the previous systematic reviews revealed that the potential studies of APP roles to be included could fall into 4 main categories: 1) medical diagnostic agreement, triaging agreement of potential orthopaedic surgical candidates or clinical recommendations between physiotherapist in APP and physicians, 2) studies on the effectiveness of treatment provided by physiotherapists in APP roles, 3) economic evaluations of treatments provided by physiotherapists in APP roles, 4) patients satisfaction with services provided by physiotherapists in APP roles. A structured data extraction form and multiple methodological quality appraisal instruments were therefore used because of the wide variety of study designs. For studies that had more than one objective, for example those that included the evaluation of treatment effect as well as an economic evaluation or a satisfaction component, more than one quality assessment tool could be used.

No universal agreement exists regarding the selection of a methodological quality appraisal instrument, although they generally conform to requirements made by recognized organizations or scientific expert teams promoting evidence based practice. Therefore we used for the diagnostic agreement studies a tool developed by one of the authors (JCM) [14]. Although this tool was initially developed to assess diagnostic test studies, it was found to be suitable for the evaluation of the diagnostic agreement studies included in this review. The quality appraisal tool rates 14 methodological items on a scale of 0–1 and an overall percentage score is calculated where a higher score indicates a better methodological quality. For the cohort studies on the effectiveness of treatment, we used another tool developed by JCM [15]. This quality appraisal tool rates 24 methodological items on a scale of 0–2 and an overall percentage score is calculated where higher scores indicate better methodological quality. Although neither tool has been formally validated, they are based on principles of evidence-based practice [16] and have been used previously in other systematic reviews [14,15].

For the economic evaluation appraisal tool we used a modified tool from the Critical Appraisal Skills Programme (CASP) developed by the Public Health Resource Unit in England, a National Health Service organisation [17]. This quality appraisal tool rates 12 methodological items and uses yes/no questions. The yes/no questions were scored as 1 or 0 and the numeric scores were used to calculate an overall percentage score. Higher scores indicate better methodological quality. The original tool was based on methodological principals for economic evaluations developed by Drummond and colleagues [18].

For the satisfaction evaluation studies, we were unable to locate in the literature any appraisal tool specifically designed to evaluate satisfaction studies. We therefore developed a tool based on general principles of evidence-based practice [16]. We also included questions regarding important satisfaction concepts [19,20]. This quality appraisal tool rates 12 methodological items scored as yes/no responses to each question. The yes/no questions were scored as 1 or 0, respectively, and an overall percentage score was then calculated. Higher scores indicate better methodological quality.

### Data analysis

After the independent evaluation of each study, pairs of raters met to compare ratings and resolve differences. A structured consensus process was used that involved: 1) re-review of the manuscripts, 2) discussion of the adherence to standards, and 3) use of an independent third evaluator if consensus was not achieved. The latter step was not required for this study, as consensus was achieved by mutual discussion of the raters. Each total score was converted into a percentage. Weighted kappa was used to calculate preconcensus inter-rater agreement on individual items and an intraclass correlation coefficient (ICC) to evaluate inter-rater reliability of the total scores. There was no formal mechanism to exclude studies on the basis of quality, but studies were rank ordered for quality.

## Results

### Overall description of included studies

**Figure 1:**
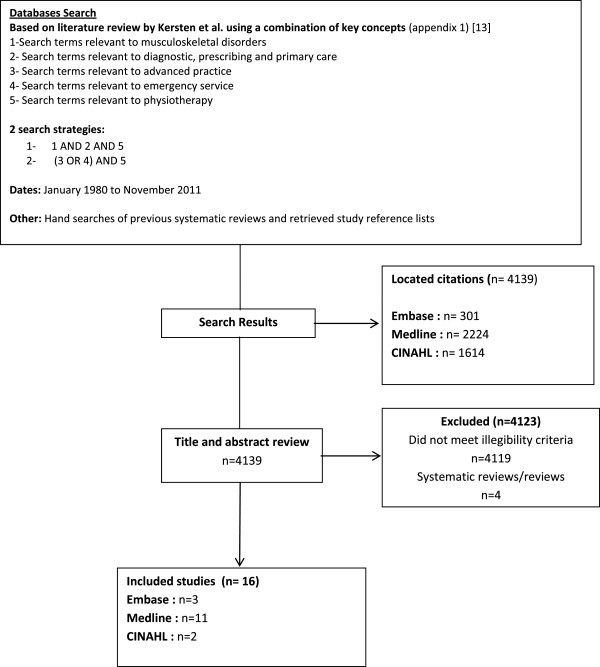
Literature search results.

The search strategies located 4139 citations; after title and abstract review, 4123 studies were excluded because they did not meet the eligibility criteria or because the located citations were reviews or systematic reviews. A total of 16 articles met all inclusion criteria and were included (Figure 1). Nine studies included in the present review had never been methodologically appraised before and seven studies had been included in previous systematic reviews (Table 1) [8,9,11].

**Table 1:** Included studies

**Authors**	**Study design**	**Setting**	**APP Role**	**Population**	n*	**Outcome measures**	**Main results by outcome measures**
Trompeter et al., 2010	Retrospective Diagnostic validity	Orthopaedic clinic (United Kingdom)	Triage of patients for orthopaedic consultation	Knee soft tissue or sports injuries	100	1- Comparison of diagnostic accuracy to arthroscopy for:	1- Sensitivity
							a. 68.1%
							b. 90.7%
						a. APP	Specificity
						b. Orthopeadic surgeon	a. 66.6%
							b. 71.4%
							Comparison in number of incorrect diagnosis:
							17/50 for APP compared to 9/50 for surgeon (p < 0.07)
						2- Identification of surgical candidates	2- Number of correctly selected surgical candidates
						a. APP	a. 47/50
						b. Orthopeadic surgeon	b. 43/50
							No significant differences between providers (p = 0.20)
MacKay, et al., 2009	Inter-rater agreement	Orthopaedic clinic (Canada)	Triage of patients for orthopaedic consultation and treatment recommendations (rehabilitation interventions)	Hip and knee arthritis	62	Agreement between APPs and Orthopaedic surgeons:	
						1- Appropriateness to be seen by surgeon	1- Level of agreement κ = 0.69
							Observed agreement 91.8%
						2- Identification of TJA surgical candidates	2- Level of agreement κ = 0.70
							Observed agreement 85.5%
Aiken et al., 2008	Inter-rater agreement	Orthopaedic clinic (Canada)	Triage of surgical candidates for TJA and treatment recommendations (rehabilitation, medication, ordering tests, referral to other providers)	Hip and knee arthritis**	38	Agreement between an APP and an Orthopaedic surgeon:	
						1- Identification of TJA surgical candidates	1- Observed agreement 100%
						2- Surgical urgency using the WCWL-HKPT tool	2- Observed agreement 64%
						3- Treatment recommendations	3- Level of agreement κ = 0.68
Aiken and McColl, 2008	Diagnostic validity/Inter-rater agreement	Orthopaedic clinic (Canada)	Diagnosis and treatment recommendations (rehabilitation, medication, ordering tests, referral to other providers, and to surgery)	Shoulder or knee musculoskeletal impairments	24	Agreement between an APP and an Orthopaedic surgeon:	
						1- Diagnostic agreement	1- Level of agreement for knee impairments κ = 0.69
							Observed agreement for knee and shoulder impairments 90%
						2- Treatment recommendations	2- Level of agreement κ = 0.52–0.87
							Observed agreement 90%
						Diagnostic accuracy of APP compared to MRI:	
						3- Diagnostic agreement	3- APP accuracy to MRI 75%
							Orthopedic surgeon accuracy to MRI 75%
O’Donoghue and Hurley-Osing, 2007	Diagnostic validity	Physiotherapy hospital department (Ireland)	Diagnosis of new patients referred by the emergency department	Acute knee injury, of less than three weeks duration	42	Diagnostic accuracy of an APP compared to MRI	
							All knee derangements, PPV = 73,2
							ACL tear, PPV = 90,4
							Meniscal tear PPV = 55.5
Moore, J. H., 2005	Retrospective Diagnostic validity	Military hospital clinic (United States)	Primary care practitioner (rehabilitation, medication, ordering tests, referral to other providers and to surgery)	Musculoskeletal complaints of the spine or extremities	560	Comparison of diagnostic accuracy to MRI for:	Observed diagnostic accuracy:
							a. 74.5% (108/145)
							b. 80.8% (139/172)
						a. APPs	c. 35.4% (86/243)
						b. Orthopeadic surgeons	Difference in diagnostic accuracy between groups:
						c. Other healthcare providers^†^	a better than c (P = 0.001)
							b better than c (P = 0.001)
							No differences between a and b (P > 0.05)
Dickens, et al., 2003	Diagnostic validity/inter-rater agreement	Orthopaedic clinic (United Kingdom)	Diagnosis and triage of surgical candidates for arthroscopy	Knee impairments excluding severe osteoarthritis	50	Agreement between APPs and an Orthopaedic surgeon:	1- Observed agreement 76.5%
						1- Diagnostic agreement	2- Diagnostic accuracy to arthroscopy:
							Sensitivity (range depending on pathology)
						2- Diagnostic accuracy to arthroscopy for:	a. 43–93%
							b. 40–100%
						a. APPs	Specificity
						b. Orthopeadic surgeons	a. 92–98%
							b. 98–100%
Sephton et al., 2010	Prospective observational cohort	Outpatient musculoskeletal clinic (United Kingdom)	Triage of patients for orthopaedic, rheumatology or pain clinic consultations (ordering tests, referral to other providers and to surgery)	Various musculoskeletal conditions	217	Treatment outcomes for patients triaged by APP at 3 months and 12 months following care (no control group):	Mean improvement in scores and 95%CI from baseline to 3 and 12 months:
						1- Pain VAS(/10)	1- 3 m: −0.72 (−1.15 to −0.29)
							12 m: −0.80 (−1.31 to −0.29)
						2- EQ-5D questionnaire (/1)	2- 3 m: 0.044 (0.001 to 0.086)
							12 m: 0.048 (0.003 to 0.093)
						3- SF-36 questionnaire (%)	3- 3 m: −0.9% (−6.3 to 4.4)
							12 m: −4.9%(−9.9 to 0.1)
						4- Perceived improvement-PIVAS scale (%)	4- 3 m : 33% (28 to 38)
							12 m: 46% (40 to 51)
						5- Deyo and Diehl Satisfaction Questionnaire (%)	
							Proportion of patients satisfied with care:
							5- 94%
Taylor et al., 2010	Prospective non-randomised controlled trial	Three emergency departments—ED (Australia)	Primary care practitioner (rehabilitation, medication and ordering tests)	Peripheral musculoskeletal injury	315	Comparison between first line APP care and usual medical care followed by physiotherapy care for ED consultation:	Differences and 95%CI between APP care and usual care:
							Time reduction with APP care:
						1- Length of stay (min)	1- 59.5 (38.4 to 80.6) min.
						2- Wait time (min)	2- 25.0 (12.1 to 38.0) min.
						3- Treatment time (min)	3- 34.9 (16.2 to 53.6) min.
							Relative Risks (APP relative to usual care):
						4- Proportion of re-presentation to ED at 1 month follow up	4- RR : 1.02 (0.51 to 2.05)
						5- Proportion of diagnostic imaging referrals	5- RR : 0.89 (0.78 to 1.02)
							Proportion of patient satisfied and relative risk (APP relative to usual care):
							6- APP care : 85%
							Usual care: 82%
						6- Patient satisfaction	RR: 1.03 (0.94 to 1.15)
Ball and Walton, 2007	Retrospective observational cohort	Emergency department (United Kingdom)	Primary care practitioner (rehabilitation, medication and ordering tests)	Closed musculoskeletal injuries to the upper or lower extremities, including fractures	643	Comparison between APPs, nurse practitioners and physicians (senior house officers, middle grade doctors and consultants):	
							1- No differences between providers (p = 0.17)
							2- No differences between providers (p = 0.99)
						1- Proportion of ordered X-rays	
							3- APP gave more advice (p < 0.007)
						2- Proportion of positive X-rays	APP prescribed fewer assistive devices (p < 0.001)
							APP referred more patients to physiotherapy (p < 0.001)
						3- Soft tissues injury treatment recommendations	Physicians prescribed more medication than other providers (p < 0.001)
McClellan et al., 2006	Prospective quasi- experimental cohort	Emergency department (United Kingdom)	Primary care practitioner (rehabilitation, medication and ordering tests)	Patients with peripheral soft tissue injuries and associated fractures	102^‡^	Comparison between APPs, nurse practitioners (NP) and physicians on treatment outcomes for patients with ankle injuries only at 4 or 16 weeks:	
					784^°^		Mean Wait and consultation times comparisons:
						1- Mean wait time for consultation (min.)	1- APPs: 43 min., NPs: 55 min., Physicians: 80 min.
							APP significantly shorter wait time than NP and physicians (p < 0.05)
						2- Mean consultation time (min.)	2- APPs: 25 min., NPs: 15 min., Physicians: 20 min.
							No significant differences in consultation time (p > 0.05)
						3- Pain VAS (/10)	Outcome of treatment for patients with ankle injuries only at 4 weeks:
						4- Function VAS (/10)	3- No significant differences between providers (p > 0.05)
						5- SF-36 (%)	4- No significant differences between providers(p > 0.05)
						Comparison between APPs, nurse practitioners and physicians care for all patients and type of injuries:	5- No significant differences between providers (p > 0.05)
						6- Patient satisfaction (%)	Proportions of patient satisfied with care (patient who strongly agreed to question: *Overall I was satisfied with the treatment received*):
							6- APPs: 54.5% NPs: 38.9%, Physicians: 35.6% (p = 0.048)
Richardson et al. 2005	RCT and cost consequence analysis	Emergency department (United Kingdom)	Primary care practitioner (rehabilitation, medication and ordering tests)	Patients with semi or non-urgent musculoskeletal conditions	766	Comparison between APP care and usual care by emergency physician on treatment outcomes at 6 months:	
							Difference and 95%CI for days to return to usual activities or work:
						1- Return to usual activities (days)	1- 12.5 added days for APP care. APP care marginally longer than usual care (p = 0.07)
						2- Return to work (days)	2- 1 added day for APP care (−3.0 to 1.0). No differences between providers (p > 0.05)
							Difference in proportions of patient satisfied with care and 95%CI:
						3- Satisfaction with care	3- 74% for usual care and 89% for APP care : 15% difference (9 to 21%)
						Economic analysis	
						4- Direct costs to healthcare system	4- No differences in costs between the two types of care (p > 0.05)
						5- Direct costs to patients	5- No differences in costs between the two types of care (p > 0.05)
						6- Indirect costs (productivity loss)	6- No differences in costs between the two types of care (p > 0.05)
Daker-White et al., 1999	RCT and cost minimisation analysis	Orthopaedic clinic (United Kingdom)	Primary care practitioner (rehabilitation, medication, ordering tests, referral to other providers and to surgery)	Patients with musculoskeletal complaints	481	Comparison between APP care and usual care by orthopeadic surgeons in training (UK junior doctors):	
							Treatment outcomes at a mean 5.6 months follow-up:
							No significant differences between providers for outcomes 1 to 8 (p > 0.05)
						Treatment outcomes at a mean 5.6 months follow-up:	
						1- Pain VAS (/10)	Use of health services:
						2- Oswestry Disability Index (%)	
						3- St-Michael's (48-0)	9- Significant difference in the proportion of patients with no test ordered (p < 0.01): 14.7% for surgeons and 47.5% for APP
						4- WOMAC (0–96)	
						5- Perceived handicap (DRP)	
						6- SF-36 (%)	
						7- Psychological status (HADS)	Significant difference in the proportion of patients with X-rays ordered (p < 0.01): 41.4% for surgeons and 13% for APP
						8- Self-efficacy	
						Use of health services	10- Significant difference in the proportion of patients who received advice and reassurance (p < 0.01): 32.5% for surgeons and 58.9% for APP
						9- Use of diagnostic tests for consult	
						10- Treatment recommendations	
						Satisfaction with care	Significant difference in the proportion of patients who received Intra-muscular injections (p < 0.01): 3.9% for surgeons and 0.5% for APP
						11- Patients	
						12- Referring general practitioners	
						Economic analysis	Significant difference in the proportion of patients who were referred for surgery (p < 0.01): 17% for surgeons and 7.1% for APP
						13- Direct costs to patients	
						14- Direct costs to healthcare system (NHS)	
							Satisfaction with care for patients and referring GP
							11- Satisfaction scores and 95%CI:
							Staff communication/attitudes (scale from 19–95) 4.6 points significant difference (2.2 to 6.8) favoring APP care
							Perceived treatment quality (scale from 13–65) 3.0 points significant difference (1.3 to 4.9) favoring APP care.
							Facilities (scale from 5–25) 0.9 point significant difference (0.3 to 1.7) favoring APP care.
							12- No significant differences between providers (p > 0.05)
							Direct costs differences
							13- No differences in costs between the two types of care (p > 0.05)
							14- Significant difference in direct hospital costs (p < 0.01):
							£498.38 for surgeon care and £255.55 for APP care.
Hockin and Bannister, 1994	Retrospective observational cohort	Orthopaedic clinic (United Kingdom)	Primary care practitioner (rehabilitation, orthotic, injection, ordering tests, referral to other providers and to surgery)	Patients with musculoskeletal complaints	189	Patient self reported global perception of improvement (%):	
						1- At the end of treatments by APP	1- 71% of patients improved by more than 40% on scale of improvement.
						2- Comparison of type of APP treatment and proportion of patients who improved:	2- More patients reported improvement with orthotics or injections than with advice and physiotherapy or surgery and referrals to other medical providers. (p < 0.05)
Kennedy et al., 2010	Cross-sectional observational study	Orthopaedic clinic (Canada)	Follow-up care after hip and knee arthroplasty	Hip and knee arthroplasty patients	123	Comparison of patients satisfaction measured by the modified VSQ-9 questionnaire:	
							Satisfaction score
						a. APP led follow-up clinic	a. 89.8%
						b. Orthopaedic surgeon led follow-up clinic	b. 87.6%
							No significant differences between providers (p = 0.34)
Campos Ayling et al. 2002	Cross-sectional observational study	Paediatric rheumatology clinic (Canada)	Review and manage independently pre-selected patients and refer to rheumatologist when tests and medication are needed	Pediatric patients with Juvenile Idiopathic Arthritis	358	Comparison of patients satisfaction measured by the modified GHAA questionnaire	
							Summary satisfaction score (5 point scale):
						a. APPs led clinic	a. 4.0 ±0.7
						b. Rheumatologists led clinic	b. 4.0 ±0.7
							No significant differences between care models (P > 0.05)

APP: advanced physiotherapy practice TJA: total joint arthroplasty WCWL-HKPT: Western Canada Wait List Project- Hip and Knee Prioritization Tool, MRI: Magnetic resonance imaging, PPV: positive predictive value, VAS: Visual analog scale, 95%CI: 95% confidence interval, ED: Emergency department, RCT: Randomized controlled trial GP: general practionner, NHS: National Health Service.

* Number of patients participating in the study.

** Majority of participants likely suffered from osteoarthritis.

^†^ Other health care providers included physicians, podiatrists, nurse practitioners and physician assistants.

^‡^ Number of patients included for the outcomes analysis on ankle injuries.

° Number of patients included for the satisfaction analysis.

Overall, seven studies were designed to evaluate either medical diagnostic agreement and accuracy, agreement on triage decisions of potential orthopaedic surgical candidates, or level of agreement for clinical recommendations between physiotherapists in advanced practice physiotherapy and physicians and two of these were retrospective in design (Table 1) [21-27].

Seven studies were cohort studies on the effectiveness of treatment provided by physiotherapists in APP roles; [13,28-33] of which two studies also included economic evaluation components [29,30], and five others evaluated patient satisfaction of services provided by APPs [13,29-32]. In terms of design, two studies were randomized controlled trials [29,30], one was a non-randomized controlled trial [32], one was a prospective quasi experimental study [13], one study was a prospective observational study [31] and two were retrospective observational studies [28,33]. An additional two cross sectional studies were specifically designed to evaluate satisfaction of services provided by APPs as their sole objective [34,35]. Settings in which these studies took place included: orthopaedic clinics (n = 8 studies), emergency departments (n = 4 studies), a military hospital clinic (n = 1 study), a specialized outpatient musculoskeletal clinic (n = 1 study), a physical therapy department (n = 1 study), and a paediatric rheumatology clinic (n = 1 study). The majority of the studies were carried out in countries with nationalized health care systems (n = 9 in the United Kingdom, n = 4 in Canada, n = 1 in Australia, n = 1 in Ireland) and one in the United States. The APP roles described varied depending on the clinical setting and country and could include: 1- communicating a medical diagnosis; 2- triaging patients to be seen by physicians or specialists for consultation or surgery; 3- ordering of diagnostic tests (imaging or laboratory) 4- conservative treatment recommendations that may include medication prescription and/or injection; 5- referral to other health care providers including to physiotherapists.

### Methodological quality of included studies and inter-rater agreement

There was a wide range in the quality of the individual studies. The study with the highest methodological quality had a score of 93% and the one with the lowest score reached only 25%; 43% of papers reached or exceeded a score of 70% on at least one of the quality rating scales (Tables 2, 3, 4, 5). Overall inter-rater reliability of the critical appraisal tools were found to be very good to excellent: 1- the diagnostic validity appraisal tool (ICC = 0.85; 95% confidence interval = 0.35–0.97); 2- for the cohort appraisal tool (ICC = 0.98; 95% confidence interval = 0.86–0.99); and 3- the satisfaction study appraisal tool (ICC = 0.89; 95% confidence interval = 0.61–0.99). Agreement between raters on individual evaluation criteria items for all three appraisal tools ranged from fair to excellent (κ = 0.4 − 1.0). For the economic appraisal tool, only two studies were appraised and therefore neither the calculation of overall ICC nor Cohen’s Kappa agreement was possible. Overall observed agreement between raters on the total of individual evaluation criteria items was 96%.

**Table 2:** Methodological quality of studies on medical diagnostic agreement and accuracy, triaging agreement of potential orthopaedic surgical candidates or clinical recommendations between physiotherapists in advanced practice physiotherapy and physicians

**Study**	**MacKay et al. 2009**	**O'Donoghue and Hurley-Osing 2007**	**Dickens et al. 2003**	**Moore, J. H. 2005**	**Trompeteret al. 2010**	**Aiken and McColl 2008**	**Aiken et al. 2008**
**Item Evaluation Criteria**							
(maximum = 1; minimum = 0)*							
1. Independent, blind comparison with a reference standard test	1	1	1	0	0	1	1
2. Reference standard/true diagnosis selected is a recognized gold standard or reasonable alternative	1	1	1	1	1	1	1
3. Reference standard applied to all patients	1	0	0	1	1	0	0
4. Actual cases include an appropriate spectrum of severity	1	1	1	1	1	1	0
5. Non-cases patients are patients who might reasonably present for differential diagnosis	1	1	1	1	1	1	1
6. Non-cases include an appropriate spectrum of patients with alternate diagnoses	1	1	1	1	1	1	0
7. Justified sample size or not less than 40 participants	0	1	1	1	0	0	0
8. Test manoeuvre described in sufficient detail to permit replication	1	1	1	1	1	0	0
9. Exact criteria for interpreting the test results provided	0	0	0	0	0	0	N/A
10. The reliability of the test procedures documented	0	1	0	0	0	0	N/A
11. Number of positive and negative results reported for both cases and non-cases	1	0	0	0	0	1	1
12. Appropriate statistics presented (sensitivity, specificity, positive/negative predictive value or likelihood ratios)	0	0	1	0	0	0	0
13. The qualifications and skills of the examiner described if the test required an element of examiner interpretation	1	1	1	1	1	0	0
14. Training, skills and experience of the examiner found to be appropriate for test interpretation	1	1	1	1	1	0	0
**Total score (%)**	71%	71%	71%	64%	57%	42%	33%
**Rank**	1	1	1	2	3	4	5

*1 indicates that criterion was fulfilled and 0 indicates that criterion was not fulfilled or not reported.

N/A=not applicable to paper. Scores obtained after consensus.

**Table 3:** Methodological quality of studies on the effectiveness of treatment provided by physiotherapists in advanced practice physiotherapy rolesphysiotherapy and physicians

**Study**	**Taylor et al. 2010 **	**Richardson et al. 2005**	**Daker-White et al. 1999**	**Sephton et al. 2010**	**McClellan et al. 2006**	**Hockinet al. 1994**	**Ballet al. 2007**
**Item Evaluation Criteria**							
(maximum = 2; minimum = 0)*							
1. Relevant background cited to establish a foundation for research question	2	0	2	2	2	2	2
2. Comparison group used	2	2	2	0	1	0	1
3. Patient status considered at more than one time point	0	2	2	2	2	1	0
4. Data collection performed prospectively	2	2	2	2	2	2	1
5. Randomization	0	2	2	0	0	0	0
6. Patients blinding	1	0	0	0	0	1	0
7. Treatment providers blinding	1	1	1	1	1	1	1
8. Independent evaluator of outcome measures	2	2	2	2	2	0	0
9. Sampling procedures minimized biases	2	2	1	1	0	1	0
10. Inclusion/exclusion criteria well-defined	2	2	2	2	1	1	1
11. Enrolment obtained to attain adequate statistical power	2	2	2	0	0	0	0
12. Appropriate retention/follow-up (>90% = 2, >70% = 1, ≤ 70% = 0)	N/A	1	1	0	0	0	N/A
13. Intervention applied according to established principles	2	1	1	1	1	1	0
14. Biases due to the treatment provider minimized	1	1	1	1	1	1	0
15. Intervention compared to an appropriate comparator	2	2	2	0	1	0	1
16. Appropriate validated primary outcome	2	1	0	1	1	1	0
17. Appropriate validated secondary outcomes	2	2	2	2	2	0	1
18. Appropriate follow-up	N/A	2	2	2	2	1	0
19. Appropriate statistical testing	2	1	2	2	2	1	2
20. Adequate power to identify treatment effects	2	1	1	1	0	1	0
21. Size and significance of treatment effect reported	2	2	2	2	1	1	0
22. Missing data accounted for and considered in analyses	1	2	0	2	0	0	0
23. Clinical and practical significance considered in interpretation of results	2	1	1	2	1	1	1
24. Conclusions and recommendations supported by the study objectives, analysis and results	2	1	2	2	2	1	1
**Total score (%)**	81%	73%	73%	63%	52%	38%	25%
**Rank**	1	2	2	3	4	5	6

*2 indicates that criterion was fulfilled, 1 indicates that criterion was partially fulfilled and 0 indicates that criterion was not fulfilled or not reported.

N/A = not applicable to paper. Scores obtained after consensus.

**Table 4:** Methodological quality of the economic analyses component for cohort studies on the effectiveness of treatment provided by physiotherapists in advanced practice physiotherapy roles

**Study**	**Daker-White et al. 1999**	**Richardson et al. 2005**
**Item Evaluation Criteria** (maximum = 1; minimum = 0)*		
1. Well-defined question posed	0	1
2. Comprehensive description of the competing alternatives	0	0
3. Evidence that the programme would be effective	1	1
4a. Identification of all important and relevant resource use and health outcome consequences for each alternative	1	0
4b. Resources measured accurately in appropriate units (hours of treatments, numbers of visits, etc.)	0	0
4c. Resources valued credibly	0	0
5. Resource use and health outcomes consequences adjusted for different times at which they occurred (discounting)	1	1
6. Incremental analysis of the consequences and costs of alternatives performed	1	0
7. Adequate sensitivity analysis performed	0	1
8. Discussion of the results includes issues that are required to inform a purchasing decision	0	0
9. Conclusions of the evaluation justified by the evidence presented	1	0
10. Applicability of results to local setting	1	1
**Total score (%)**	50%	42%

*1 indicates that criterion was fulfilled and 0 indicates that criterion was not fulfilled or not reported.

N/A = not applicable to paper. Scores obtained after consensus.

**Table 5:** Methodological quality of satisfaction studies or cohort studies with a satisfaction component for services provided by physiotherapists in advanced practice physiotherapy roles

**Study**	**Kennedy et al. 2010**	**Taylor et al. 2010**	**Daker-White et al. 1999**	**Sephton et al. 2010**	**Campos-Ayling et al. 2002**	**McClellan et al. 2006**	**Richardson et al. 2005**
**Item Evaluation Criteria** (maximum = 1; mimimum = 0)*‡							
1. Relevant background cited to establish a foundation for research question	1	1	1	1	0	1	0
2. Adequate description of the study setting and patients characteristics	1	1	1	1	1	0	1
3. Inception cohort sampled	1	1	1	1	1	1	1
4. Data collection process administered by independent evaluators	1	0	1	1	1	0	1
5. Respondents informed that their results are anonymous or not shared with treatment providers	1	0	0	0	0	0	0
6. Standardized satisfaction tool/measure used with known validity and reliability; Item	1	0	0	1	1	0	0
7. Timing of data collection sufficiently close to care treatment/encounter as to minimise recall bias;	0	1	0	1	0	1	1
8. Accounted for missing data;	1	0	0	0	0	0	0
9. ≥ 80% of eligible patients sampled	1	1	1	0	0	0	0
10. Clearly defined measurements of components of satisfaction:							
a. Affability/Patients centeredness and interpersonal interactions with providers	1	1	1	1	1	1	0
b. Process (accessibility, availability, efficiency of care)	1	N/A	1	N/A	1	N/A	0
c. Perceived competency of professionals	1	1	1	1	1	1	0
d. Satisfaction with outcomes	1	1	0	N/A	1	1	N/A
11. Appropriate statistical test(s) performed;	1	1	1	0	1	1	1
12. Conclusions and clinical recommendations supported by the study objectives, analysis and results	1	1	1	1	1	1	0
**Total score (%)**	93%	71%	71%	69%	67%	57%	36%
**Rank**	1	2	2	3	4	5	6

*1 indicates that criterion was fulfilled and 0 indicates that criterion was not fulfilled or not reported.

N/A = not applicable to paper. Scores obtained after consensus.

#### Medical diagnostic, triage and clinical recommendations agreement studies

**Description and main findings of studies** Agreement between APPs and orthopaedic surgeons regarding medical diagnosis, and triage of patients for conservative care or review by surgeons for potential surgical candidates was found to range from good to excellent (range κ = 0.69 to 1.00) [21,22,24] and treatment recommendations agreement ranged from fair to very good (range κ = 0.52 to 0.70) [22,24]. Four studies evaluated the diagnostic accuracy of APPs where the gold standard was diagnostic imaging or surgery; one study reported the accuracy of APPs to be good compared to MRI [26] and three studies reported the accuracy of APPs to be good and comparable to the diagnostic accuracy of orthopaedic surgeons [22,23,27]. In another study, diagnostic accuracy of APPs was found to be similar to that of the orthopaedic surgeons, and significantly better than that of other healthcare providers including physicians, podiatrists, nurse practitioners and physician assistants [25].

**Methodological quality** 3 of the 7 studies on medical diagnostic agreement and accuracy, triaging agreement of potential orthopaedic surgical candidates or clinical recommendations for conservative management between physiotherapists in advanced practice physiotherapy and physicians reached a methodological score of 71% or greater (Table 2). For 3 of the 7 studies, the reference standard used (Item 3) for comparison of the APPs’ ability to communicate a diagnosis or triage patients was the orthopaedic surgeons’ clinical diagnosis. In the other studies that used diagnostic imaging or surgical findings as the reference standard, often not all patients underwent these reference procedures, which may have introduced bias. In all studies, the exact criteria or tests used (item 9) regarding the APPs’ diagnostic process (subjective and objective evaluation) were not reported, and the reliability of the diagnostic process (item 10) was not assessed in 5 of the 7 studies. Finally, in 6 of the 7 studies the appropriate statistical measures such as specificity, sensitivity, predictive values or likelihood ratios were missing (item 12).

#### Studies on the effectiveness of treatment for APP care

**Description and main findings of studies** Four of the seven studies compared APP care to usual care either in an emergency department or an orthopaedic clinic. Three of these studies, using various outcome measures, did not report any differences in treatment effectiveness between APPs and physicians or other providers [13,29,32]. The study by Richardson et al. reported a tendency for a delayed return to usual activities for participants treated by APPs compared to participants treated by a physicians in an emergency department, but that trend was not seen in days needed to return to work following initial injury [30]. Regarding the type of treatment recommendations, APPs significantly gave more advice to patients [28,29], prescribed less medication and injections [29], and fewer assistive devices [28]. APPs referred more patients to physiotherapy [28] and fewer to surgery than physicians [29]. Wait time and treatment time for consultation were also compared in two studies [13,32]. Taylor and Norman found that total length of stay (wait time and treatment time) for APP care in emergency departments was significantly shorter than usual care with a physician [32]. However, the study of McClellan et al. [13] did not find any significant differences in treatment times for APP care compared to care with a physician or with a nurse practitioner. In terms of health services use, one study reported that APP working in an orthopaedic clinic ordered significantly less diagnostic tests (laboratory and imaging) than surgeons in training (UK junior doctors) [29] and in another study there were no significant differences in the number of X-rays ordered between APPs and physicians [28].

**Methodological quality** 3 of the 7 cohort studies on the effectiveness of treatment for APP care had a methodological score of 73% or more (Table 3). Only 2 out of 7 studies were randomized controlled trials (item 5) [29,30]. Patients blinding was absent for all 5 studies whose design and intervention would allow it (item 6) [13,28-31]. Because of the nature of the studies included here, treatment providers blinding was not possible for any of the included studies (item 7). For 2 studies the appropriate retention or follow-up proportion was below the recommended 90% [29,30] and below 70% for three other studies (item 12) [13,31,33]. Six out seven studies did not adequately report the established principles for the interventions, especially in the case of defining the APPs treatment approach (item 13) [13,28-31,33]. For all 7 studies, minimal attention was directed either in the methods or discussion to the potential for treatment provider biases, although the two reviewers believed the risks for biases were potentially low for 6 of the studies and were scored 1 out 2 (item 14) [13,29-33]. Selection of an appropriate primary outcome measure was problematic for 6 of the 7 studies (item 16) [13,28-31,33], but selection of secondary outcome measures was found adequate for 5 of these studies (item 17). Only 1 study reported adequate power to identify treatment effect (item 20) [32] and only 2 studies reported adequate strategies to account for missing data in their analyses (item 22) [30,31].

#### Economic evaluations of treatments provided by physiotherapists in APP

**Description and main findings of studies** The study by Richardson and colleagues did not find any significant differences between APP care and usual care by a physician working in an emergency department, in terms of direct costs to the healthcare system or indirect costs to the patients [30]. Likewise, the study by Daker-White et al. evaluating costs in an orthopaedic clinic, did not find any significant differences between APP care and usual care by surgeons in training (UK junior doctor) in terms of indirect costs to the patients, however the direct medical costs were significantly lower for APP care compared to usual care by junior doctors [29].

**Methodological quality** The two economic evaluations of treatments provided by APPs had methodological scores of 42% and 50% (Table 4) Comprehensive description of the competing alternatives was lacking for the two studies, especially for the description of the APP interventions (items 3). It was unclear in both articles how important resources were measured and valued (Items 4b and 4c) and discussion of the results did not include issues required to inform a purchasing decision (item 9).

#### Patients’ satisfaction of services provided by physiotherapists in APP

**Description and main findings of studies** Seven studies evaluated patients’ satisfaction of services provided by physiotherapists in APP roles. Three studies took place in emergency departments, two in an orthopaedic clinic, one in a specialized outpatient musculoskeletal clinic and one in a paediatric rheumatology clinic. Patient’s satisfaction levels regarding APP services were high for all seven studies and three studies comparing APP care to usual medical care showed significantly higher satisfaction for the APP care [13,29,30]. Three other studies did not find a significant difference between the two types of care [32,34,35].

**Methodological quality** The quality of studies varied greatly (range 36-93%) and three studies had a mean methodological score over 70% (Table 5) [29,32,35]. Only one study reported having informed participants that their results were anonymous and not shared with treatment providers (item 5) [35]. Four of the studies did not use a standardized satisfaction tool/measure with known validity and reliability (item 6) [13,29,30,32]. Only one study accounted for missing data in their analyses (item 8) [35] and only three studies had a follow-up proportion of more than 80% (item 9) [29,32,35].

## Discussion

### Main findings

This systematic review focused on evaluating the expanding role of advanced practice/extended scope physiotherapists in the management of patients with musculoskeletal disorders. Sixteen studies met our inclusion criteria and were methodologically appraised. The methodological quality varied greatly but was only adequate for a minority of studies as 7 out 16 of the papers exceeded a score of 70% on at least one methodological tool (some studies were appraised with more than one tool).

The scope of the APP roles varied somewhat depending on the country, the setting (primary care, emergency department or orthopaedic clinic) and the precise population under care, but generally included: communicating a medical diagnosis, triaging patients, ordering of diagnostic tests, conservative treatment recommendations and referral to other health care providers. Overall results and conclusions made by the authors of the studies included in this review supported the role of APP in terms of treatment effectiveness and patients were as satisfied, or more satisfied, with this new model of care than usual care by physicians. Only one study reported that APP treatment in an emergency department led to a prolonged time before patients return to usual activities and advised against such a model of care, however this difference was not seen in time to return to work [30]. In terms of diagnostic agreement and validity, the ability of APPs to communicate a diagnosis or triage patients was generally found to be as good as orthopaedic surgeons [21-25]. Of interest, one study found the diagnostic validity of APPs was better than that of non-orthopaedic physicians [25]. In terms of health services use, in two studies APPs did not order more X-rays than doctors [28,29]. Also, in terms of wait time, in two studies taking place in emergency departments, the mean consultation time for APPs was found to be the same [13] or shorter than usual care by a physician [32]. These data again support the efficiency of the APP model of care. In terms of economic costs, although both studies were found to have poor methodological quality for the economic component analysis, direct costs to patients [29,30] and indirect costs were also similar between the two types of care [30]. In terms of directs costs to the health care system, no significant differences were found between the two types of care in the study by Richardson et al. [30] However, direct medical costs were lower for the APP care compared to the junior doctor care in the study by Daker-White et al. [29]. These findings suggest that APP care may cost less than usual care. Overall, our findings highlight the need for more methodologically sound studies. Although some studies are of limited quality, their findings are consistent and suggest that APP care may be as (or more) beneficial than usual care by physicians for patients with musculoskeletal disorders, in terms of diagnosis, treatment effectiveness, use of healthcare resources, economic costs and patient satisfaction.

### Comparison with previous reviews

Compared to the previous systematic review by McPherson and colleagues [11] on extended roles for health professionals, the current review, which included recent new evidence, suggests that physiotherapists can indeed learn specific advanced skills outside their routine scope of practice and apply them. Previously, McPherson and colleagues did not provide any specific conclusions regarding the APP role in their initial review [11]. Their second review, published in 2007, was specific to the APP role. The authors concluded that their review demonstrated overwhelming support for APP roles, especially in terms of improving access to care and patient satisfaction [8]. However, the review by Kilner on the effect of emergency department physiotherapy services concluded that the available evidence did not support the use of physiotherapists in emergency departments. Their review focused not only on APPs but also included studies where physiotherapists without additional scopes of practice worked in the emergency department. This review initially included four studies that were also included in the present review, however, the author’s conclusions were ultimately based on only two of these studies [9]. Although there is disagreement regarding the benefits of APPs when comparing these reviews, Kilner and the team of Kersten and colleagues, all outlined the same issues in regards to the methodological quality of studies on APP roles; all authors concluded that studies on APP roles were generally methodologically weak and that more methodologically sound studies were needed to draw any definitive conclusions on the benefits of APP care [8,9,11].

### Methodological quality and implication for future research

Similar to previously published reviews, the methodological quality of many of the studies included in the present systematic review continue to be a problem. To allow for more robust results and conclusions, future studies on APP roles must be designed with better methodological rigour. In terms of diagnostic agreement or validity, the use of an unbiased comparator is necessary and was lacking in many studies. Comparing the diagnostic accuracy of physiotherapists to only one medical practitioner may not be optimal. Future studies should include more than one practitioner who specializes in that field and, when relevant, could also use a diagnostic imaging reference test for comparison of all participants included in the study. The reporting of results using a two by two table and associated statistical measures (sensitivity, specificity, positive/negative predictive value or likelihood ratios) should also be systematically done. Similarly, for triage agreement or treatment recommendation studies, the comparison of the physiotherapists’ recommendations to more than one medical practitioner is advised. Patient blinding was not done for any of the cohort studies investigating treatment effectiveness included in the present review. Although there are methodological challenges in trying to blind patients to the identity of the practitioners, we advocate it can be done and should be done to reduce potential bias. In terms of definition of treatment, especially for APP care, better description of the exact treatment options for the providers should be described. The use of a validated, reliable and responsive primary outcome measure was lacking in almost all of the studies included in the current review. The strategy for handling missing data should be clear and a priori sample size calculation should also be done. Regarding economic evaluation studies, the reporting of all resources and costs measured should be thorough; the methods to value them should be clear and sensitivity analyses should be performed. For studies investigating patient satisfaction with APP care, participants should be informed that their results are anonymous or not shared with treatment providers. We also recommend the use of validated satisfaction questionnaires. In our review, many included studies did not use a validated tool and it was unclear which components of patient satisfaction were evaluated. As outlined by other authors, it should be clearly reported which of these important aspects of care are being measured: 1- interactions with providers; 2- process with care (accessibility and availability of service); 3- perceived competency of providers and 4-satisfaction with outcomes [19,20].

Another important methodological element that future studies need to address is the number of physiotherapists in APP roles that are included for evaluation. We realize that advanced practice physiotherapy remains an emerging role in many countries and settings, often resulting in a very limited number of individuals practicing in these new roles, but future studies would benefit by including more physiotherapists, as well as by including more than one study setting. Including multiple individuals and settings would greatly increase the external validity or generalizability of the results and conclusions. Another limitation of the current literature is the lack of reporting of the participating physiotherapists’ background and training.

### Strengths and limitations of the present review

The search strategy was very broad and most likely insured that all relevant literature was included. The search strategy used was based on a previous review and was updated and performed by a professional health sciences documentalist, one of the co-authors (OH) [8]. We identified four important research areas (diagnostic accuracy, treatment effectiveness, economic efficiency and patient satisfaction) relevant to the evaluation of APP care, and we used four specific methodological appraisal tools that allowed us to summarize the findings and quality of the available literature in each of these areas. One of the four diagnostic study appraisal tools we used was initially developed to assess diagnostic tests. Hence, some of the methodological items may have been more difficult to interpret in the context of an agreement study and may have led to more variability between raters. The inter-rater agreement was high nonetheless but the resulting confidence interval was large. For the three other methodological appraisal tools, the inter-observer agreement was high and confidence intervals were found to be relatively narrow.

One of the limitations of our review is that, for the evaluation of the satisfaction studies, we developed a new tool. This was done because we were unable to locate in the literature any appraisal tool specifically designed to evaluate such studies. Although it was based on general principles of evidence-based practice [16] and included questions regarding important satisfaction concepts, this tool has not been formally validated [19,20]. More research is therefore needed to fully validate its use. However for five included studies, this tool was used in conjunction with the cohort appraisal tool. Interestingly, for four of the five studies, the relative methodological rankings were the same for both tools. Only the study by Richardson et al. was ranked differently, moving from second place with the cohort tool to the sixth place with the satisfaction tool [30]. Another limitation of this review is that, although it is generally recognized that APP care will impact access to care by reducing wait time for a consult or for surgery [8], the included studies in our review did not present any data regarding that aspect of APP care. We were therefore unable to make any specific conclusions as to the effects of APP roles on access to care. Finally, because of the various outcome measures, different settings, interventions and populations, this systematic review did not allow for the pooling of study results to do a meta-analysis. Nonetheless, we believe that our results add to the body of knowledge on APP care and will help clinicians, investigators and stakeholders in understanding and making decisions regarding the development and evaluation of such models of care.

## Conclusions

This review highlights the need for more methodologically sound studies to evaluate the effectiveness of emerging advanced practice/extended scope roles for physiotherapists. Despite the lack of methodological rigor of the studies reviewed, findings provide consistent, albeit low grade, evidence that for patients with musculoskeletal disorders, APP care may be as beneficial (or more so) than usual care by physicians in terms of diagnostic accuracy, treatment effectiveness, use of healthcare resources, economic costs and patient satisfaction.

## Appendix

The initial search strategy used a combination of MeSH terms (subject headings) and keywords for professions (physiotherapy) and interventions (APP). The search strategy was further expended  to also include: 1- keywords related to diagnostic ability and/or diagnostic agreement between APP and other health providers or diagnostic imaging; 2- keywords related to emergency medicine and 3- the search was limited to a population with musculoskeletal disorders (Table 6).

**Table 6:** Detailed search strategy with keywords and descriptors


**1- Search terms used to identify resources relevant to musculoskeletal disorders**	
***Musculoskeletal diseases ***^***M(exp)***^	***Musculoskeletal disease ***^***E(exp)***^
***Musculoskeletal system ***^***M(exp), E(exp)***^	***Back pain ***^***M(exp)***^
***Low back pain ***^***E***^	
**2- Search terms used to identiffy resources relevant to diagnostic, prescribing and primary care**	
***Diagnosis***^***M, E***^	***Decision Making***^***M,E***^
***Diagnosis, Differential***^***M, E***^	***Magnetic resonance imaging***^***M, E***^
Diagnos* - diagnos(is/es/tic/tics/tician)	***Primary health care***^***M, E,C***^
***Diagnosis, musculoskeletal ***^***C***^	***Prescriptive autority ***^***C***^
***Disability Evaluation***^***M***^	
**3- Search terms used to identify resources relevant to advanced practice**	
advanc* ADJ4 practi*	***professional role ***^***M***^
clinical specialist*	***professional standards ***^***E***^
consultant*	profession* boundar*
**consultants **^***M,C***^	**reprofessionali?ation**
cross boundar*	**prompt access**
current role*	role* boundar*
direct access*	role* ADJ1 chang* - role(s) chang(ed/es/ing)
**direct access **^***C***^	***role change ***^***C,E***^
**early ADJ1 access**	role* ADJ1 collaborati* - role(s) collaborati(ve/on)
emerging role*	role* ADJ1 cross* - role(s) cross(ing/over(s))
enhan* ADJ4 practice* - enhan(ced/cing/sion(s)) practice(s)	role* ADJ1 defin* - role(s) defin(e/ed/ing/ition(s))
enhan* ADJ4 scope* - enhanc(ed/ing/ement(s)) scope(s)	role* demarcation*
existing role*	role* ADJ1 develop*
existing scope*	role* ADJ4 enhan* - role(s) enhanc(ed/ing/ement(s))
expan* ADJ4 practice* - expan(ded/ding/sion(s)) practice(s)	role* ADJ4 expan* - role(s) expan(ded/ding/sion(s))
expan* ADJ4 scope* - expan(ed/ing/sion(s)) scope(s)	role* ADJ4 exten* - role(s) exten(ded/ding/sion(s))
ext* ADJ4 scope* - extra / exten(ded/ding/sion(s)) scope(s)	role* ADJ4 interdisciplin* - role(s) interdisciplin(e/ary)
exten*ADJ4 practice* - exten(ded/ding/sion(s)) practice(s)	role* ADJ1 interprofessional*
**initial ADJ1 assessment**	role* ADJ4 modern* - role(s) modern(ise(d)/ising/isation)
int??disciplinary competenc* - (intra/inter)disciplinary c	role* ADJ4 overlap* - role(s) overlap(s/ped/ping)
int??disciplinary practice* - (intra/inter)disciplinary p	role* ADJ1 professional*
**interdisciplinary collaboration**	role* ADJ 4 redefin* - role(s) redefin(e/ed/ing/ition(s))
***interprofessional relations***^***M***^	role* ADJ1 shar* - role(s) shar(ed/es/ing)
interprofessional relation*	role* ADJ1 shift* - role(s) shift(s/ed/ing)
joint practice*	**scope of practice**
led ADJ4 clinic*	***scope of practice***^***C***^
led ADJ4 service*	shar* ADJ4 competenc* - shar(ed/ing competenc(e/y/ies)
multi* task*	shift* ADJ4 boundar*
new role*	skill* ADJ4 interdisciplin*
new scope*	skill* ADJ4 overlap* - skill(s) overlap(s/ped/ping)
physician exten*	skill* ADJ4 shar*
physician* assist*	specialist practitioner*
physiotherap* practitioner*	traditional role*
physical therap* practitioner*	transdisciplinary practice*
**primary contact**	**triage **^***M, E***^
profession* ADJ4 autonomy	**triage**
***professional autonomy ***^***M***^	
**4- Search terms used to identify resources relevant to emergency service**	
***Emergency Service, Hospital ***^***M, E***^	
***Emergency Service ***^***C***^	
**5- Search terms used to identify resources relevant to physiotherapy**	
exercise therap* - exercise therap(y/ies/ist(s))	***physical therapy service ***^***C***^
***exercise therapy ***^***M(exp)***^	***physical therapy (specialty) ***^***M***^
kinesiotherap* - kinesiotherap(y/ist(s))	***physical therapy modalities ***^***M(exp)***^
***kinesiotherapy ***^***E(exp)***^	**physio**
manual therap*	**physios**
***manual therapy ***^***C(exp)***^	physiotherap*
physical therap*- physical therap(y/ist(s)/ies)	**physiotherapist **^***E***^
***physical therapists ***^***C***^	***physiotherapy***^***, E(exp)***^
***physical therapy ***^***C(exp)***^	***physiotherapy practice ***^***E***^

M=Medline, C=Cinahl, E=Embase

exp: expanded, adj: adjacent

Modified search strategy based on litterature review of Kersten et al. [8].

## Competing interests

The authors declare that they have no competing interests.

## Authors’ contributions

FD participated in the design of the review, the literature search, the extraction of data and the methodological appraisal of studies. He performed the statistical analyses, led the interpretation of results and drafted the manuscript. LJW participated in the design, the literature search, the extraction of data, the methodological appraisal of studies, the interpretation of results and the writing of the manuscript. JSR participated in the design, the literature search, the extraction of data, the methodological appraisal of studies and the writing of the manuscript. FC and JCM participated in the design, the interpretation of results and the writing of the manuscript. OH participated in the literature search, the extraction of data and the writing of the manuscript. All authors read and approved the final version of the paper.

## Pre-publication history

The pre-publication history for this paper can be accessed here:

http://www.biomedcentral.com/1471-2474/13/107/prepub
